# Potential effects of traditional massage on spasticity and gross motor function in children with spastic cerebral palsy: A randomized controlled trial

**DOI:** 10.12669/pjms.35.5.478

**Published:** 2019

**Authors:** Qamar Mahmood, Shaista Habibullah, Muhammad Naveed Babur

**Affiliations:** 1 Dr. Qamar Mahmood, National Institute of Rehabilitation Medicine, Islamabad Pakistan; 2 Dr. Shaista Habibullah, PhD, National Institute of Rehabilitation Medicine, Islamabad Pakistan; 3 Prof. Dr. Muhammad Naveed Babur, PhD, Dean, Faculty of Rehabilitation Sciences, Isra University, Pakistan

**Keywords:** Cerebral palsy, Motor activity, Massage, Modified ashworth scale, Muscle spasticity

## Abstract

**Objective:**

To evaluate the effects of traditional massage (TM) on spasticity and gross motor function in children with cerebral palsy (CP).

**Methods:**

This randomized control trial (RCT) was conducted with recruitment of 86 children (Dropped out= 11; Analyzed= 75) with spastic CP (diplegia) allocated randomly through sealed envelope method to intervention and control group with ages between 2-10 years from September 2016 to August 2018. Both groups received conventional physical therapy (CPT) once daily, five times a week for a period of three months. However, intervention group received TM additionally. Modified Ashworth Scale (MAS), Gross Motor Function Measure (GMFM-88) and Gross Motor Function Classification System (GMFCS) were used to evaluate spasticity and gross motor function at the beginning, after six and 12 weeks of intervention. Data were compared and analyzed through SPSS-20.

**Results:**

Mean age in control and intervention group was 6.81±2.31 and 7.05±2.47 years respectively. No statistically significant differences in MAS, GMFM and GMFCS scores were found at base line. The children in intervention group showed statistically significant reduction in MAS scores in all four limbs after six and 12 weeks of intervention (p < 0.05) in comparison with the control group. However, GMFM scores and GMFCS levels did not change significantly in intervention group as compared to control group.

**Conclusion:**

It is concluded that TM can effectively reduce the spasticity, does not have harmful effects, so can be administered safely by mothers at home and making it suitable for the management of spastic CP. However, in order to achieve better gross motor function, it should be practiced in conjunction with CPT, functional skills and task oriented approaches.

## INTRODUCTION

Cerebral palsy (CP) is considered as the most common type of physical disability presenting in pediatric population across the globe.^1^ Worldwide population-based studies have reported its prevalence estimates which range from 1.5 to 4 per 1,000 live births.^2^ CP produces disorders of tone, movement and posture. Abnormal uncontrolled high tone stiffens the body parts (termed as spasticity), accounts for 65-70% of all tone disorders and results in muscle stiffness, joint contractures and deformities which in turn restricts the affected person’s activity and participation leading to compromised quality of life.^3^

Many interventions are in use to manage the disabling and lifelong effects of CP. In a systematic review, Novak et al. categorized 64 such interventions which are in current use for the management of CP and amongst these massage is also included.^4^ Practice of massage shows different and unique forms across the globe for which masseurs are educated, trained and certified.^5^ However traditional massage (TM) which is practiced in its own way in each society without special training, education or certification. In Pakistan, TM is mostly performed by mothers or care givers in a way quite different to Swedish massage which is considered as a standard amongst the professionals.

Disability remains common amongst poor countries with low socio-economic status and hence multiplies its burden many folds. This increases the need to devise such interventions which are locally available, affordable, and doable by the poor population. World Health Organization has always stressed the use of such interventions to handle the ever increasing burden of disability in poor countries.^6^ In such a scenario TM which is already being practiced in the homes with no financial implications on caregivers becomes a viable option.

Previous studies to find the effectiveness of massage in the management of spasticity has produced conflicting results. In one randomized controlled trial (RCT), Hernandez concluded reduction of spasticity and improvement in functional skills in intervention group which received Swedish massage for three months.^7^ On the other hand, Alizad 8 and Rasool 9 in their RCTs on the use of massage did not find such effectiveness. No study has been conducted so far in Pakistan to find the effectiveness of the traditional Pakistani massage on spasticity and gross motor function of children with spastic cerebral palsy although its technique of application differs to already established approaches. Moreover difference of opinion also exists amongst different knowledgeable and credible health care professionals regarding use and effects of massage in the management of spasticity which puts the parents in a dilemma. This situation led to conduction of a RCT to evaluate the effects of TM on the spasticity and gross motor function and to investigate whether TM has any influence on both these variables.

## METHODS

This study (RCT) conducted at the Physiotherapy department of National Institute of Rehabilitation Medicine (NIRM) Islamabad from August 2016 to September 2018 used non-probability, purposive sampling technique to recruit the participants and allocated them to intervention and control groups randomly through sealed envelope method. Children with ages between 02-10 years having diagnosis of spastic CP (diplegia: spastic paralysis of both lower limbs to major extent with mild involvement of both upper limbs) were included. All children with musculoskeletal contractures, moderate to severe mental retardation, attention deficit hyperactive disorder (ADHD), behavioral disorders and uncontrolled seizures were excluded.

Sample size was calculated by taking a confidence interval of 95% with a power of 80% and using OpenEpi version three software which provided the result as 42 per each group. Mean and standard deviation taken for control and intervention group for this RCT were 61.85±18.2 & 49.97±20.2 respectively with a difference of 11.88 between the groups. Elgawish et al. conducted a study which was taken as a reference for this purpose.^10^ When the enrolment completed, 86 participants with spastic diplegia were recruited.

Both groups received conventional physical therapy (CPT) for 30 minutes once daily, five times a week for a period of 12 weeks. Intervention group also received TM additionally, a session of 30 minutes before CPT. A detailed training program and guidance about CPT and TM was provided to the mothers / caregivers till the satisfaction of concerned physical therapist. After this training, caregivers provided CPT and TM at homes. CPT comprised strengthening exercises, stretching exercises and optimal positioning.^11^ Spastic muscles were stretched up to the level of mild discomfort with hold time of thirty seconds and repetition of five times. All weak muscle were provided with resistive exercises ten times in one session. Parents were directed to make sitting of their children with open legs on some bench/block in such a way that heels should touch the ground. They were advised to keep the child’s thoraco-lumber spine straight (in order to avoid posterior pelvic tilt) and to avoid crossed legs and W sitting postures on the floor. Standing position was advised against a wall with open legs (in moderate abduction and external rotation) daily for 15 minutes.

TM for the purpose of this study was defined as gently rubbing the parts of the body with oil in proximal to distal direction on all four extremities while on back and front of trunk area, from center to periphery. This rubbing was ensured gentle, superficial and longitudinal in nature. Deep pressures, circular movements and stroking were avoided which are mostly practiced in Swedish massage. Each extremity, front and back of trunk area was provided with TM for five minutes each.

Ethical Review Committee of Isra University, Islamabad accorded ethical approval (Letter/ Student/2015/10/27/1505. Oct-27-2015 ). Informed consent was secured from the parents. They were ensured that this study is safe involving minimal risk and no harm to participants. Data were collected by qualified, well trained and experienced physical therapist using a structured questionnaire which comprised demographic and baseline characteristics shown in Table-I, Modified Ashworth Scale (MAS) to assess the severity of spasticity subjectively, Gross Motor Function Measure (GMFM-88) for in depth assessment of gross motor ability and Gross Motor Function Classification System (GMFCS) to know the mobility status of the participants at baseline, after six and 12 weeks of intervention. MAS, GMFM-88 and GMFCS are most commonly used standardized tools in children with spastic CP. Outcome assessors was blinded of group identification of children during all successive assessments.

**Table I:** Demographic Characteristics of study population.

Demographic Characteristics	Study Population (n= 75)	Control Group (n= 37)	Intervention Group (n= 38)	P-value
Age in years	6.93±2.37	6.81±2.10	7.05±2.47	0.663a
Gender (Numbers)				0.390b
Male	47(63%)	25(68%)	22(58%)	
Female	28(37%)	12(32%)	16(42%)	
Birth Place				0.178b
Govt. Facility	31(41%)	14(38%)	17(45%)	
Pvt. Facility	29(39%)	12(32%)	17(45%)	
Home	15(20%)	11(30%)	4(10%)	
Nature of Pregnancy				0.741b
Full term	56(75%)	27(73%)	29(76%)	
Pre-Mature	19(25%)	10(27%)	9(24%)	
P-Natal Complications				0.986b
Temperature	11(15%)	4(11%)	7(18%)	
Delayed Cry	40(53%)	22(60%)	18(48%)	
Ventilation	4(5%)	2(5%)	2(5%)	
Others	20(27%)	9(24%)	11(29%)	
GMFCS Levels				0.621a
Ambulatory (I-III)	54(62%)	27(63%)	27(61%)	
Non-Ambulatory (IV-V)	21(28%)	10(27%)	11(29%)	

a: p-value calculated with parametric test, b: p-value calculated with non-parametric tests.

### Statistical analysis

Data were analyzed using SPSS-20 software. Data revealed that both groups were quite identical in characteristics at the start of the study (Table-I). Paired sample T-test and Independent Sample T-Test were used to compare mean differences within the groups and between the groups respectively.

**Fig.1:**
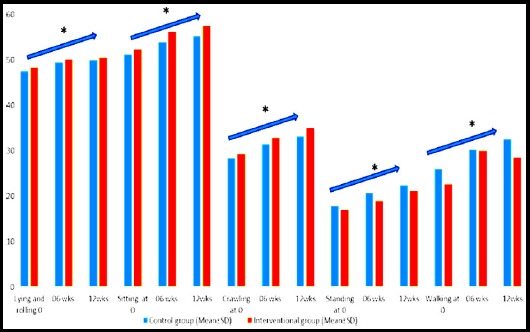
GMFM sub-domains scores.

## RESULTS

Out of 86 enrolled participants, Six (7%) from control group while five (6%) from intervention group dropped out leaving 37(43%) and 38(44%) in both groups respectively. So finally data of 75 participants were analyzed. Main reasons of this drop out were lack of motivation, transport difficulties, telecommunication issues and surgical correction of two participants during intervention period. No statistically significant differences in MAS, GMFM and GMFCS scores were found at base line (Tables I-II).

**Table II:** Comparison of MAS grades between and within groups.

MAS		Control Group (Mean±SD)	Intervention Group (Mean±SD)	P-value
All Four Limbs	Baseline	0.91±0.45	0.87±0.42	0.72
	After 6 Wks	0.75±0.46	0.54±0.40	0.04
		0.001a	0.001a	
	After 12 Wks	0.57±0.43	0.36±0.32	0.01
		0.001b	0.001b	

a: p value (within group) baseline to 06 weeks, Wks: Weeks, b: p-value (within group) baseline to 12 weeks.

On comparison of MAS grades between the groups in all four limbs, results showed statistically significant reduction in intervention group after six & 12 weeks (p < 0.05) of intervention. Comparative analysis within the groups showed significant reduction in MAS grades after six and 12 weeks of intervention in both groups. In case of MAS grades, lower is better and optimal which denotes reduction in spasticity (Table-II).

On comparison of GMFCS scores between the groups, there were no statistically significant differences after six and 12 weeks (p > 0.05) of intervention. However comparative analysis within the groups showed significant decline in GMFCS scores after six weeks and 12 weeks of intervention in intervention group & after 12 weeks in control group (p < 0.05) which means better mobility because for GMFCS scores, lower is better and optimal (Table-III).

**Table III:** Comparison of GMFCS scores between and within groups.

GMFCS	Control Group (Mean±SD)	Intervention Group (Mean±SD)	P-value
Baseline	2.86±0.82	2.92±0.91	0.780
After 6 Wks	2.78±0.88	2.76±0.91	0.921
	0.083a	0.057a	
After 12 Wks	2.62±1.06	2.58±0.95	0.855
	0.002b	0.001b	

a: p value (within group) baseline to 06 weeks, Wks: Weeks, b: p-value (within group) baseline to 12 weeks.

On comparison of GMFM scores between the groups, there were no statistically significant differences after six and 12 weeks (p > 0.05) of intervention. However comparative analysis within the groups showed statistically significant increase in GMFM scores after six and 12 weeks of intervention in both groups which means better gross motor activity because for GMFM scores, higher is better and optimal (Table-IV).

**Table IV:** Comparison of GMFM total scores between and within groups.

GMFM Total score	Control Group (Mean±SD)	Intervention Group (Mean±SD)	P-value
Baseline	64.76±22.16	64.55±18.95	0.966
After 6 Wks	70.76±21.42	71.24±16.85	0.914
	0.001a	0.001a	
After 12 Wks	74.27±21.50	74.32±16.58	0.992
	0.001b	0.001b	

a: p value (within group) baseline to 06 weeks, Wks: Weeks, b: p-value (within group) baseline to 12 weeks.

## DISCUSSIONS

Spasticity results in restricted movements, development of musculo-skeletal contractures, impaired mobility, difficulty in performing activities of daily living, participation restrictions and decreased quality of life.^12^ In the management of CP, reduction in spasticity and improvement in motor activity always remain the prime goals of any intervention. The main focus of the current study was to examine the effects of TM on spasticity and gross motor function of children with spastic CP. Results showed statistically significant decrease in spasticity in the intervention group as compared to control group in all four limbs after six and 12 weeks of intervention. In one RCT, Hernandez et al. reported the decline of spasticity in the intervention group which received Swedish massage for 12 weeks in comparison to control group which was provided with reading activities. However they observed that this reduction was significant in upper limbs only. In lower limbs although it showed decreasing tendency but not up to a significant level.^7^ This study partially supports the current study with respect to significant reduction in spasticity in which it took place in all four limbs.

In another RCT, Alizad et al. used Swedish massage by certified experts along with occupational therapy techniques for twelve weeks and observed that although the decreasing trend of spasticity was noted in both groups but no significant differences were found between the groups. However, on within group comparison he reported significant reduction of spasticity in muscles of trunk in intervention group.^8^ The findings of this study partially supports the current study in which reduction of spasticity was significant in all four limbs on comparison between and within both groups.

In one study carried out at the same Institute, Rasool applied localized deep friction massage on gastrocnemius and soleus along with CPT for six weeks and observed no significant reduction in spasticity between the groups. However, on comparison within groups, significant reduction in spasticity was observed in intervention group.^9^ Macgregor in a case series of five adolescents with spastic diplegia using Swedish massage for 12 weeks observed no significant reduction of spasticity.^13^

Although the studies of Hernandez^7^, Rasool^9^, Alizad^8^ and Macgregor^13^ in which certified professionals provided the massage, partially supports the current study where significant reduction in spasticity took place in all four limbs on comparison between and within both groups but its strength lies in the fact that instead of experts, it was the parents who provided the TM and CPT at homes. This shows that home exercise program can be equally effective if carried out with proper training, supervision and guidance. This aspect might be helpful in decreasing the financial burden faced by parents and society due to costly and lifelong rehabilitation process of children with CP. In a double blind RCT, Novak et al concluded that home exercise programs are quite effective and produce satisfactory results hence should be prescribed by health professionals.^14^

As far as improvement in gross motor function is concerned, results of current study showed no significant increase in GMFM scores & and no significant decrease in GMFCS scores in intervention group as compared to control group at six and 12 weeks of intervention. Rasool reported the same findings in his study but he used a nine point self-structured questionnaire for assessment of motor function whose reliability and validity was not established.^9^ However within group analysis of the current study showed significant increase in GMFM scores & significant decrease in GMFCS scores in both groups in which CPT was being provided as a common therapeutic technique. Multiple publications in healthcare literature support the findings of current study and reflect the effectiveness of physical therapy in reducing spasticity, improving gross motor function and mobility of the children with CP.^4^,^15^,^16^

Hernandez et al. reported significant improvement in fine motor and gross motor activities in intervention group in arms after 12 weeks of intervention with Swedish massage as compared to control group. The improvement noted in this case might be due to the fact that he used Developmental Programming for Infants and Young Children (DPIYC) as an assessment tool which does not provide in depth assessment of motor activity. Out of six subscales of DPIYC, only one is related to gross motor assessment. On the other hand GMFM-88 is a comprehensive assessment tool of gross motor function which has been used in the current study and consists of total 88 items to be assessed in five domains of lying/rolling, sitting, crawling, standing and walking.^7^

The salient finding of the current study is that TM reduced spasticity to a significant level in intervention group as compared to control group but similar change did not happen in gross motor function. This might be due to the fact that spasticity behaves differently in resting positions versus activity performance. It is well documented fact that spasticity increases with emotions, fear, lack of balance, in anti-gravity postures and intend to perform new skills.^15^,^17^ As spasticity is assessed by using MAS scale in resting, cool and calm supine position, its intensity categorized there is actually not reflected when the child tries to perform new, complex and coordinated movements as practiced during gross motor assessment using GMFM-88. Therefore TM should be practiced in conjunction with therapeutic measures, functional skills and task oriented approaches to get combined benefit of reduction of spasticity and enhancement of gross motor function.

### Limitations of study

As parents were providing the interventions at home, the frequency, intensity and continuity of the treatment could not be monitored directly. However indirect monitoring was ensured to its maximum. The present study was conducted on children aged 2-10 years and is not generalizable for adolescents and adults with spastic CP.

## CONCLUSION

It is concluded that TM can effectively reduce the spasticity, does not have harmful effects so it can be administered safely by mothers at home, making it suitable for the long-term management of spastic CP. However in order to achieve better gross motor function it should be practiced in conjunction with CPT, functional skills and task oriented approaches.

### Recommendations

Further research is recommended to explore following effects in detail.

To examine the difference in behavior, pattern and progression of spasticity in resting versus non-resting positions/postures with different interventions.

To examine the behavior of spasticity with different study designs and interventions to know how and when reduced spasticity can be transformed into enhanced motor function.

### Author`s Contribution

**QM** conceived, designed, did data collection & initial writing of manuscript.

**SH** did critical review, proof reading and final approval of manuscript.

**MNB** did overall guidance and supervision.
